# Supplementary material for: Next-Generation Sequencing of a Large Gene Panel for Outcome Prediction of Bariatric Surgery in Patients with Severe Obesity
Source: J Clin Med. 2022 Dec 19;11(24):7531. doi: 10.3390/jcm11247531 (PMC9783894; doi:10.3390/jcm11247531)

# - Supplementary Material –

*List of diagnostic and candidate genes*

**Table S1.** Diagnostic genes sequenced in the present study. Gene, OMIM ID, associated pathology and reference as from OMIM or literature are reported.

| Gene | OMIM ID | Gene-phenotype relationship | Reference |
|---|---|---|---|
| ADRA2 | 104210 | Atypical familial partial lipodystrophy | [42] |
| AGPAT2 | *603100 | Lipodystrophy, congenital generalized, type 1 | 608594 |
| AKT2 | 164731 | AKT2-linked lipodystrophy | [43] |
| ALDH18A1 | 138250 | Cutis laxa, autosomal recessive, type IIIA | 219150 |
| ALMS1 | 606844 | Alström syndrome | 203800 |
| ARL6 | 608845 | Bardet-Biedl syndrome 3 | 600151 |
| BBIP1 | 613605 | Bardet-Biedl syndrome 18 | 615995 |
| BBS1 | 209901 | Bardet-Biedl syndrome 1 | 2099009 |
| BBS10 | 610148 | Bardet-Biedl syndrome 10 | 615987 |
| BBS12 | 610683 | Bardet-Biedl syndrome 12 | 615989 |
| BBS2 | 606151 | Bardet-Biedl syndrome 2 | 615981 |
| BBS4 | 600374 | Bardet-Biedl syndrome 4 | 615982 |
| BBS5 | 603650 | Bardet-Biedl syndrome 5 | 615983 |
| BBS7 | 607590 | Bardet-Biedl syndrome 7 | 615984 |
| BBS9 | 607968 | Bardet-Biedl syndrome 9 | 615986 |

| | | | |
|---|---|---|---|
| BSCL2 | *606158 | Lipodystrophy, congenital generalized, type 2 | 269700 |
| C8orf37 | 614477 | Bardet-Biedl syndrome 21 | 617406 |
| CARTPT | 602606 | Obesity | [44] |
| CAV1 | *601047 | Lipodystrophy, familial partial, type 7 | 606721 |
| CAVIN1 | *603198 | Lipodystrophy, congenital generalized, type 4 | 613327 |
| CEP19 | 615586 | CEP19 deficiency obesity | 615703 |
| CEP290 | 610142 | Bardet-Biedl syndrome 14 | 615991 |
| CIDEC | 612120 | Lipodystrophy, familial partial, type 5 | 615238 |
| DYRK1B | 604556 | Abdominal Obesity-Metabolic Syndrome | 615812 |
| FTO | *610966 | Obesity | 612460 |
| GHR | *600946 | Hypercholesterolemia | 143890 |
| GNAS | 139320 | McCune-Albright syndrome | 174800 |
| HDAC8 | 300269 | Cornelia de Lange syndrome | 300882 |
| IFT172 | 607386 | Bardet-Biedl syndrome | 615630 |
| IFT27 | 615870 | Bardet-Biedl syndrome 19 | 615996 |
| INPP5E | 613037 | MORM syndrome | 610156 |
| INSR | 147670 | INSR deficiency hyperinsulinism | 610549 |
| KSR2 | 610737 | Obesity and insulin resistance | [45] |
| LEP | 164160 | Obesity from congenital leptin deficiency | 614962 |
| LEPR | 601007 | Obesity from leptin receptor deficiency | 614963 |
| LIPE | 151750 | Lipodystrophy, familial partial, type 6 | 615980 |
| LMNA | 150330 | Lipodystrophy, familial partial, type 2 | 151660 |
| LZTFL1 | 606568 | Bardet-Biedl syndrome 17 | 615994 |
| MC3R | 155540 | Susceptibility to severe obesity | 602025 |
| MC4R | 155541 | Obesity from melanocortin receptor deficiency 4 | 618406 |
| MCHR1 | 601751 | Obesity | 601751 |
| MEGF8 | 604267 | Carpenter syndrome 2 | 614976 |
| MFN2 | 608507 | Obesity | [46] |
| MKKS | 604896 | Bardet-Biedl syndrome | 605231 |

| | | | |
|---|---|---|---|
| MKS1 | 609883 | Bardet-Biedl syndrome 13 | 615990 |
| NCOA2 | *601993 | Obesity phenotype | 601993 |
| NPC1 | *607623 | Niemann-Pick disease, type C1 | 257220 |
| NR0B2 | 604630 | Obesity | 601665 |
| NSD1 | 606681 | Sotos syndrome 1 | 117550 |
| PALB2 | *610355 | Accumulation of adipose tissue | [47] |
| PCSK1 | 162150 | Prohormone convertase 1 deficiency obesity | 600955 |
| PDX1 | *600733 | MODY, type IV | 606392 |
| PHF6 | 300414 | Borjeson-Forssman-Lehmann syndrome | 301900 |
| PLIN1 | 170290 | Lipodystrophy, familial partial, type 4 | 613877 |
| PNPLA2 | *609059 | Neutral lipid storage disease with myopathy | 610717 |
| POMC | 176830 | Obesity from pro-opiomelanocortin deficiency | 609734 |
| POU1F1 | 173110 | Lipedema | [48] |
| PPARG | 601487 | Severe obesity | 601665 |
| PPP1R3A | 600917 | Severe insulin resistance, digenic | 125853 |
| RAB23 | 606144 | Carpenter's syndrome | 201000 |
| RYR1 | *180901 | Central core disease | 117000 |
| SDC3 | *186357 | Obesity, association with | 601665 |
| SDCCAG8 | 613524 | Bardet-Biedl syndrome 16 | 615993 |
| SH2B1 | 608937 | Obesity | [49] |
| SIM1 | 603128 | Obesity | [50] |
| TBL1XR1 | 608628 | Pierpont syndrome | 602342 |
| TRIM32 | 602290 | Bardet-Biedl syndrome 11 | 615988 |
| TTC8 | 608132 | Bardet-Biedl syndrome 8 | 615985 |
| UCP3 | 602044 | Severe obesity and insulin resistance | 601665 |
| VPS13B | 607817 | Cohen's syndrome | 216550 |
| WDPCP | 613580 | Bardet-Biedl syndrome | 615992 |
| ZMPSTE24 | *606480 | Mandibuloacral dysplasia with type B lipodystrophy | 608612 |

**Table S2.** Candidate genes sequenced in the present study. Gene, description and OMIM ID are reported.

| Gene | Description | OMIM ID |
|---|---|---|
| *A2M* | ALPHA-2-MACROGLOBULIN | *103950 |
| *A2ML1* | ALPHA-2-MACROGLOBULIN-LIKE | *610627 |
| *ABCC6* | ATP-BINDING CASSETTE, SUBFAMILY C, MEMBER 6 | *603234 |
| *ABCG4* | ATP-BINDING CASSETTE, SUBFAMILY G, MEMBER 4; ABCG4 | *607784 |
| *ABCG1* | ATP-BINDING CASSETTE, SUBFAMILY G, MEMBER 1 | *603076 |
| *ACBD7* | ACYL-CoA BINDING DOMAIN CONTAINING | - |
| *ACSL1* | ACYL-CoA SYNTHETASE LONG CHAIN FAMILY, MEMBER 1 | *152425 |
| *ACVR1C* | ACTIVIN A RECEPTOR, TYPE IC | *608981 |
| *ADCY3* | ADENYLATE CYCLASE | *600291 |
| *ADIG* | ADIPOGENIN | *611396 |
| *ADIPOQ* | ADIPOCYTE-, C1q-, AND COLLAGEN DOMAIN-CONTAINING | *605441 |
| *ADRB2* | BETA-2-ADRENERGIC RECEPTOR | 109690 |
| *ADRB3* | BETA-3-ADRENERGIC RECEPTOR; | *109691 |
| *AEBP1* | AE-BINDING PROTEIN 1 | *602981 |
| *AGRP* | AGOUTI-RELATED NEUROPEPTID | *602311 |
| *AKR1B1* | ALDO-KETO REDUCTASE FAMILY 1, MEMBER B1 | *103880 |
| *AKR1B10* | ALDO-KETO REDUCTASE FAMILY 1, MEMBER B10 | *604707 |
| *AKR1B15* | ALDO-KETO REDUCTASE FAMILY 1, MEMBER B15 | *616336 |
| *AKR1C1* | ALDO-KETO REDUCTASE FAMILY 1, MEMBER 1 | *600449 |
| *AKR1C2* | ALDO-KETO REDUCTASE FAMILY 1, MEMBER C2 | *600450 |
| *AKR1C3* | ALDO-KETO REDUCTASE FAMILY 1, MEMBER C3 | *603966 |
| *AKR1C4* | ALDO-KETO REDUCTASE FAMILY 1, MEMBER C4 | *600451 |
| *AKR1E2* | ALDO-KETO REDUCTASE FAMILY 1, MEMBER E2 | *617451 |
| *AKT2* | AKT SERINE/THREONINE KINASE 2 | *164731 |
| *ANGPTL4* | ANGIOPOIETIN-LIKE 4 | *605910 |
| *ANK2* | ANKYRIN | *06410 |
| *ANKRD26* | ANKYRIN REPEAT DOMAIN-CONTAINING PROTEIN 26 | *610855 |
| *ANXA1* | ANNEXIN A1 | *151690 |

| | | |
|---|---|---|
| *APOA1* | APOLIPOPROTEIN A-I | *107680 |
| *APOE* | APOLIPOPROTEIN E | *107741 |
| *ARNTL* | ARYL HYDROCARBON RECEPTOR NUCLEAR TRANSLOCATOR-LIKE PROTEIN | *602550 |
| *ARRDC3* | ARRESTIN DOMAIN-CONTAINING 3 | *612464 |
| *ATG12* | AUTOPHAGY-RELATED 12 | *609608 |
| *ATXN1* | ATAXIN 1 | *601556 |
| *BAIAP3* | BAI1-ASSOCIATED PROTEIN 3 | *604009 |
| *BDNF* | BRAIN-DERIVED NEUROTROPHIC FACTOR | *113505 |
| *BECN2* | BECLIN 2 | *615687 |
| *BRD2* | BROMODOMAIN-CONTAINING PROTEIN 2 | *601540 |
| *BRS3* | BOMBESIN-LIKE RECEPTOR 3 | *300107 |
| *BSCL2* | BSCL2 GENE | *606158 |
| *CADM2* | CELL ADHESION MOLECULE 2 | *609938 |
| *CAMKK2* | CALCIUM/CALMODULIN-DEPENDENT PROTEIN KINASE KINASE 2, BETA | *615002 |
| *CAV1* | CALCIUM/CALMODULIN-DEPENDENT PROTEIN KINASE KINASE 2, BETA; CAMKK2 | *601047 |
| *CAVIN1* | CAVEOLAE-ASSOCIATED PROTEIN 1 | *603198 |
| *CCBE1* | COLLAGEN AND CALCIUM-BINDING EGF DOMAIN-CONTAINING PROTEIN 1 | *612753 |
| *CD300E* | CD300E ANTIGEN | *609801 |
| *CDKN1A* | CYCLIN-DEPENDENT KINASE INHIBITOR 1A | *116899 |
| *CDKN1B* | CYCLIN-DEPENDENT KINASE INHIBITOR 1B | *600778 |
| *CIDEA* | CELL DEATH-INDUCING DFFA-LIKE EFFECTOR | *604440 |
| *CLOCK* | CIRCADIAN LOCOMOTOR OUTPUT CYCLES KAPUT | *601851 |
| *CNR1* | CANNABINOID RECEPTOR 1 | *114610 |
| *CNTNAP2* | CONTACTIN-ASSOCIATED PROTEIN-LIKE 2 | *604569 |
| *COL3A1* | COLLAGEN, TYPE III, ALPHA-1 | *120180 |
| *CPE* | CARBOXYPEPTIDASE E | *114855 |
| *CPEB4* | CYTOPLASMIC POLYADENYLATION ELEMENT-BINDING PROTEIN 4 | |
| *CRY2* | CRYPTOCHROME 2 | *603732 |
| *CYP19A1* | CYTOCHROME P450, FAMILY 19, SUBFAMILY A, POLYPEPTIDE | *107910 |
| *DNAAF1* | DYNEIN, AXONEMAL, ASSEMBLY FACTOR 1 | *613190 |
| *DYRK1A* | DUAL-SPECIFICITY TYROSINE PHOSPHORYLATION-REGULATED KINASE 1A | *600855 |

| | | |
|---|---|---|
| *EBF1* | EARLY B-CELL FACTOR 1 | *164343 |
| *ELN* | ELASTIN | *130160 |
| *ENPP1* | ECTONUCLEOTIDE PYROPHOSPHATASE/PHOSPHODIESTERASE 1 | *173335 |
| *EPAS1* | ENDOTHELIAL PAS DOMAIN PROTEIN 1 | *603349 |
| *ESR1* | ESTROGEN RECEPTOR 1 | *133430 |
| *ESRRA* | ESTROGEN-RELATED RECEPTOR, ALPHA | *601998 |
| *FABP2* | FATTY ACID-BINDING PROTEIN 2 | *134640 |
| *FABP4* | FATTY ACID-BINDING PROTEIN 4 | *600434 |
| *FFAR4* | FREE FATTY ACID RECEPTOR 4 | *609044 |
| *FGF21* | FIBROBLAST GROWTH FACTOR 21 | *609436 |
| *FLT4* | FMS-LIKE TYROSINE KINASE 4 | *136352 |
| *FOXC2* | FORKHEAD BOX C2 | *602402 |
| *FOXO1* | FORKHEAD BOX O1 | *136533 |
| *GATA2* | GATA-BINDING PROTEIN 2 | *137295 |
| *GCKR* | GLUCOKINASE REGULATORY PROTEIN | *600842 |
| *GDF15* | GROWTH/DIFFERENTIATION FACTOR 15 | *605312 |
| *GDF3* | GROWTH/DIFFERENTIATION FACTOR 3 | *606522 |
| *GFRAL* | GDNF FAMILY RECEPTOR ALPHA-LIKE PROTEIN | *617837 |
| *GHRL* | GROWTH HORMONE SECRETAGOGUE RECEPTOR LIGANDMOTILIN-RELATED PEPTIDE | *605353 |
| *GHSR* | GROWTH HORMONE SECRETAGOGUE RECEPTOR | *601898 |
| *GJA1* | GAP JUNCTION PROTEIN, ALPHA-1 | *121014 |
| *GPD1* | GLYCEROL-3-PHOSPHATE DEHYDROGENASE 1 | *138420 |
| *GPR26* | G PROTEIN-COUPLED RECEPTOR 26 | *604847 |
| *GPR82* | G PROTEIN-COUPLED RECEPTOR 82 | *300748 |
| *GPRC6A* | G PROTEIN-COUPLED RECEPTOR, FAMILY C, GROUP 6, MEMBER A | *613572 |
| *GRAMD2B* | GRAM Domain Containing 2B | - |
| *GRB14* | GROWTH FACTOR RECEPTOR-BOUND PROTEIN 14 | *601524 |
| *GRIK1* | GLUTAMATE RECEPTOR, IONOTROPIC, KAINATE 1 | *138245 |
| *GRPR* | GASTRIN-RELEASING PEPTIDE RECEPTOR | *305670 |
| *GSDMB* | GASDERMIN B | *611221 |
| *GSK3A* | GLYCOGEN SYNTHASE KINASE 3-ALPHA | *606784 |

| | | |
|---|---|---|
| *GSK3B* | GLYCOGEN SYNTHASE KINASE 3-BETA | *605004 |
| *GUCY2C* | GUANYLATE CYCLASE 2C | *601330 |
| *H6PD* | HEXOSE-6-PHOSPHATE DEHYDROGENASE | *138090 |
| *HDAC4* | HISTONE DEACETYLASE 4 | *605314 |
| *HGF* | HEPATOCYTE GROWTH FACTOR | *142409 |
| *HIF1A* | HYPOXIA-INDUCIBLE FACTOR 1, ALPHA SUBUNIT | *603348 |
| *HIPK2* | HOMEODOMAIN-INTERACTING PROTEIN KINASE 2 | *606868 |
| *HMGA2* | HIGH MOBILITY GROUP AT-HOOK 2 | *600698 |
| *HOXA1* | HOMEOBOX A1 | *142955 |
| *HOXA10* | HOMEOBOX A10 | *142957 |
| *HOXA2* | HOMEOBOX A2 | *604685 |
| *HOXA4* | HOMEOBOX A4 | *142953 |
| *HOXA5* | HOMEOBOX A5 | *142952 |
| *HOXA7* | HOMEOBOX A7 | *142950 |
| *HOXB4* | HOMEOBOX B4 | *142965 |
| *HOXB8* | HOMEOBOX B8 | *142963 |
| *HOXC13* | HOMEOBOX C13 | *142976 |
| *HOXC4* | HOMEOBOX C4 | *142974 |
| *HOXC8* | HOMEOBOX C8 | *142970 |
| *HOXD4* | HOMEOBOX D4 | *142981 |
| *IFI35* | INTERFERON-INDUCED PROTEIN 3 | *600735 |
| *IFT74* | INTRAFLAGELLAR TRANSPORT 74 | *608040 |
| *IL18* | INTERLEUKIN 18 | *600953 |
| *IL6* | INTERLEUKIN 6 | *147620 |
| *IL6R* | NTERLEUKIN 6 RECEPTOR | *147880 |
| *INSIG2* | INSULIN-INDUCED GENE 2 | *608660 |
| *IRS1* | INSULIN RECEPTOR SUBSTRATE | 147545 |
| *IRX3* | IROQUOIS HOMEOBOX PROTEIN 3 | *612985 |
| *ITGA9* | INTEGRIN, ALPHA-9 | *603963 |
| *KAT5* | LYSINE ACETYLTRANSFERASE 5 | *601409 |
| *KDM3A* | LYSINE DEMETHYLASE 3A | *611512 |

| | | |
|---|---|---|
| *KIRREL3* | KIRRE-LIKE NEPHRIN FAMILY ADHESION MOLECULE 3 | *607761 |
| *KLF16* | KRUPPEL-LIKE FACTOR 16 | *606139 |
| *KRAS* | KRAS PROTOONCOGENE | *190070 |
| *LPIN1* | LIPIN 1 | *605518 |
| *LRP2* | LOW DENSITY LIPOPROTEIN RECEPTOR-RELATED PROTEIN 2 | *600073 |
| *LRRC8A* | LEUCINE-RICH REPEAT-CONTAINING PROTEIN 8 | *608360 |
| *LYPLAL1* | LYSOPHOSPHOLIPASE-LIKE 1 | *616548 |
| *MAPK11* | MITOGEN-ACTIVATED PROTEIN KINASE 11 | *602898 |
| *MAPK12* | MITOGEN-ACTIVATED PROTEIN KINASE 12 | *602399 |
| *MAPK13* | MITOGEN-ACTIVATED PROTEIN KINASE 13 | *602899 |
| *MAPK14* | MITOGEN-ACTIVATED PROTEIN KINASE 14 | *600289 |
| *MC1R* | MELANOCORTIN 1 RECEPTOR | *155555 |
| *MED13* | MEDIATOR COMPLEX SUBUNIT 13 | *603808 |
| *MEIS1* | MEIS HOMEOBOX 1 | *601739 |
| *MLXIPL* | MLX-INTERACTING PROTEIN-LIKE | *605678 |
| *MMP19* | MATRIX METALLOPROTEINASE 19 | *601807 |
| *MRAP2* | MELANOCORTIN 2 RECEPTOR ACCESSORY PROTEIN 2 | *615410 |
| *MSX2* | MSH HOMEOBOX 2 | *123101 |
| *NBEA* | NEUROBEACHIN | *604889 |
| *NCOA1* | NUCLEAR RECEPTOR COACTIVATOR 1 | *602691 |
| *NCOA3* | NUCLEAR RECEPTOR COACTIVATOR 3 | *601937 |
| *NDN* | NECDIN | *602117 |
| *NEGR1* | NEURONAL GROWTH REGULATOR 1 | *613173 |
| *NEIL1* | ENDONUCLEASE VIII-LIKE 1 | *608844 |
| *NF1* | NEUROFIBROMIN 1 | *613113 |
| *NGEF* | NEURONAL GUANINE NUCLEOTIDE EXCHANGE FACTOR | *605991 |
| *NMU* | NEUROMEDIN U | *605103 |
| *NPY* | NEUROPEPTIDE Y | *162640 |
| *NPY4R* | PANCREATIC POLYPEPTIDE RECEPTOR 1 | *601790 |
| *NR1D1* | NUCLEAR RECEPTOR SUBFAMILY 1, GROUP D, MEMBER | *602408 |
| *NR2F2* | NUCLEAR RECEPTOR SUBFAMILY 2, GROUP F, MEMBER 2 | *107773 |

| | | |
|---|---|---|
| *NR2F6* | NUCLEAR RECEPTOR SUBFAMILY 2, GROUP F, MEMBER 6 | *132880 |
| *NR3C1* | NUCLEAR RECEPTOR SUBFAMILY 3, GROUP C, MEMBER 1 | *138040 |
| *NTRK2* | NEUROTROPHIC TYROSINE KINASE, RECEPTOR, TYPE 2 | *600456 |
| *OGG1* | 8-OXOGUANINE DNA GLYCOSYLASE | *601982 |
| *OMA1* | OMA1 ZINC METALLOPEPTIDASE | *617081 |
| *OSBPL8* | OXYSTEROL-BINDING PROTEIN-LIKE PROTEIN 8 | *606736 |
| *PANX1* | PANNEXIN 1 | *608420 |
| *PAX6* | PAIRED BOX GENE 6 | *607108 |
| *PBX1* | PRE-B-CELL LEUKEMIA TRANSCRIPTION FACTOR 1 | *176310 |
| *PDE11A* | PHOSPHODIESTERASE 11A | *604961 |
| *PDE3B* | PHOSPHODIESTERASE 3B | *602047 |
| *PER1* | PERIOD CIRCADIAN REGULATOR 1 | *602260 |
| *PIGC* | PHOSPHATIDYLINOSITOL GLYCAN ANCHOR BIOSYNTHESIS CLASS C PROTEIN | *601730 |
| *PIK3CA* | PHOSPHATIDYLINOSITOL 3-KINASE, CATALYTIC, ALPHA | *171834 |
| *PITPNM1* | PHOSPHATIDYLINOSITOL TRANSFER PROTEIN, MEMBRANE-ASSOCIATED, 1 | *608794 |
| *PNPLA2* | PATATIN-LIKE PHOSPHOLIPASE DOMAIN-CONTAINING PROTEIN 2 | *609059 |
| *PPARGC1A* | PEROXISOME PROLIFERATOR-ACTIVATED RECEPTOR-GAMMA, COACTIVATOR 1, ALPHA | *604517 |
| *PPARGC1B* | PEROXISOME PROLIFERATOR-ACTIVATED RECEPTOR-GAMMA, COACTIVATOR 1, BETA | *608886 |
| *PRDM16* | PR DOMAIN-CONTAINING PROTEIN 16 | *605557 |
| *PRDM2* | PR DOMAIN-CONTAINING PROTEIN 2 | *601196 |
| *PRKAA1* | PROTEIN KINASE, AMP-ACTIVATED, CATALYTIC, ALPHA-1 | *602739 |
| *PRKAA2* | PROTEIN KINASE, AMP-ACTIVATED, CATALYTIC, ALPHA-2 | *600497 |
| *PRKAB1* | PROTEIN KINASE, AMP-ACTIVATED, NONCATALYTIC, BETA-1 | *602740 |
| *PRKAB2* | PROTEIN KINASE, AMP-ACTIVATED, NONCATALYTIC, BETA-2 | *602741 |
| *PRKACA* | PROTEIN KINASE, cAMP-DEPENDENT, CATALYTIC, ALPHA | *601639 |
| *PRKACB* | PROTEIN KINASE, cAMP-DEPENDENT, CATALYTIC, BETA | *176892 |
| *PRKAG1* | PROTEIN KINASE, AMP-ACTIVATED, NONCATALYTIC, GAMMA-1 | *602742 |
| *PRKAG2* | PROTEIN KINASE, AMP-ACTIVATED, NONCATALYTIC, GAMMA-2 | *602743 |
| *PRKAG3* | PROTEIN KINASE, AMP-ACTIVATED, NONCATALYTIC, GAMMA-3 | *604976 |
| *PRKAR1A* | PROTEIN KINASE, cAMP-DEPENDENT, REGULATORY, TYPE I, ALPHA | *188830 |
| *PRKAR2A* | PROTEIN KINASE, cAMP-DEPENDENT, REGULATORY, TYPE II, ALPHA | *176910 |

| | | |
|---|---|---|
| *PRKAR2B* | PROTEIN KINASE, cAMP-DEPENDENT, REGULATORY, TYPE II, BETA | *176912 |
| *PROX1* | PROSPERO-RELATED HOMEOBOX 1 | *601546 |
| *PRRC2A* | PROLINE-RICH COILED-COIL PROTEIN 2A | *142580 |
| *PTN* | PLEIOTROPHIN | *162095 |
| *PYY* | PEPTIDE YY | *600781 |
| *RARB* | RETINOIC ACID RECEPTOR, BETA | *180220 |
| *RB1* | RB TRANSCRIPTIONAL COREPRESSOR 1 | *614041 |
| *RBP4* | RETINOL-BINDING PROTEIN 4 | *180250 |
| *RETN* | RESISTIN | *605565 |
| *RGS2* | REGULATOR OF G PROTEIN SIGNALING 2 | *600861 |
| *RREB1* | RAS-RESPONSIVE ELEMENT BINDING PROTEIN | *602209 |
| *RSC1A1* | REGULATORY SOLUTE CARRIER PROTEIN, FAMILY 1, MEMBER 1 | *601966 |
| *RSPO3* | R-SPONDIN 3 | *610574 |
| *SCD* | STEAROYL-CoA DESATURASE | *604031 |
| *SCPEP1* | SERINE CARBOXYPEPTIDASE I | - |
| *SFRP1* | SECRETED FRIZZLED-RELATED PROTEIN 1 | *604156 |
| *SFRP5* | SECRETED FRIZZLED-RELATED PROTEIN 5 | *604158 |
| *SIRT1* | SIRTUIN 1 | *604479 |
| *SIRT6* | SIRTUIN 6 | *606211 |
| *SKP2* | S-PHASE KINASE-ASSOCIATED PROTEIN 2 | *601436 |
| *SLC13A5* | SOLUTE CARRIER FAMILY 13 (SODIUM-DEPENDENT CITRATE TRANSPORTER), MEMBER 5 | *608305 |
| *SLC2A4* | SOLUTE CARRIER FAMILY 2 (FACILITATED GLUCOSE TRANSPORTER), MEMBER 4 | *138190 |
| *SLC35D3* | SOLUTE CARRIER FAMILY 35, MEMBER D3 | *612519 |
| *SLCO4C1* | SOLUTE CARRIER ORGANIC ANION TRANSPORTER FAMILY, MEMBER 4C1 | *609013 |
| *SNAP25* | SYNAPTOSOMAL-ASSOCIATED PROTEIN, 25-K | *600322 |
| *SNRPN* | SMALL NUCLEAR RIBONUCLEOPROTEIN POLYPEPTIDE | *182279 |
| *SREBF1* | STEROL REGULATORY ELEMENT-BINDING TRANSCRIPTION FACTOR 1 | *184756 |
| *STAB1* | STABILIN 1 | *608560 |
| *STAT3* | SIGNAL TRANSDUCER AND ACTIVATOR OF TRANSCRIPTION 3 | *102582 |
| *STAT5A* | SIGNAL TRANSDUCER AND ACTIVATOR OF TRANSCRIPTION 5A | *601511 |
| *STAT5B* | SIGNAL TRANSDUCER AND ACTIVATOR OF TRANSCRIPTION 5B | *604260 |

| | | |
|---|---|---|
| *STRA6* | STIMULATED BY RETINOIC ACID 6 | *610745 |
| *SYPL2* | SYNAPTOPHSIN LIKE | - |
| *TAF7L* | TATA BOX-BINDING PROTEIN-ASSOCIATED FACTOR 7-LIKE | *300314 |
| *TBC1D1* | TBC1 DOMAIN FAMILY, MEMBER 1 | *609850 |
| *TBC1D4* | TBC1 DOMAIN FAMILY, MEMBER 4 | *612465 |
| *TBX15* | T-BOX TRANSCRIPTON FACTOR 15 | *604127 |
| *TGFB1* | TRANSFORMING GROWTH FACTOR, BETA-1 | *190180 |
| *TGM2* | TRANSGLUTAMINASE 2 | *190196 |
| *TMEM18* | TRANSMEMBRANE PROTEIN 18 | *613220 |
| *TNXB* | TENASCIN XB | *600985 |
| *TRH* | THYROTROPIN-RELEASING HORMONE | *613879 |
| *TRIM72* | TRIPARTITE MOTIF-CONTAINING PROTEIN 72 | *613288 |
| *TTR* | TRANSTHYRETIN | *176300 |
| *TUB* | TUB BIPARTITE TRANSCRIPTION FACTOR | *601197 |
| *TYK2* | TYROSINE KINASE 2 | *176941 |
| *UBE2E2* | UBIQUITIN-CONJUGATING ENZYME E2E 2 | *602163 |
| *UCP1* | UNCOUPLING PROTEIN 1 | *113730 |
| *UCP2* | UNCOUPLING PROTEIN 2 | *601693 |
| *VEGFA* | VASCULAR ENDOTHELIAL GROWTH FACTOR A | *192240 |
| *VEGFC* | VASCULAR ENDOTHELIAL GROWTH FACTOR | *601528 |
| *WDR13* | WD REPEAT-CONTAINING PROTEIN 13 | *300512 |
| *WNT10B* | WINGLESS-TYPE MMTV INTEGRATION SITE FAMILY, MEMBER 10B | *601906 |
| *WNT11* | WINGLESS-TYPE MMTV INTEGRATION SITE FAMILY, MEMBER 1 | *603699 |
| *WNT4* | WINGLESS-TYPE MMTV INTEGRATION SITE FAMILY, MEMBER 4 | *603490 |
| *YWHAZ* | TYROSINE 3-MONOOXYGENASE/TRYPTOPHAN 5-MONOOXYGENASE ACTIVATION PROTEIN, ZETA ISOFORM | *601288 |
| *ZEB1* | ZINC FINGER E BOX-BINDING HOMEOBOX 1 | *189909 |
| *ZMPSTE24* | ZINC METALLOPROTEINASE STE2 | *606480 |
| *ZNF423* | ZINC FINGER PROTEIN 423 | *604557 |
| *ZNRF3* | ZINC FINGER AND RING FINGER PROTEIN | *612062 |

**Table S3.** Genetic variants identified in the diagnostic gene panel. MAF = Minor allele frequency in gnomAD. Transcript isoforms: *BBS1*, NM_024649.5; *BBS2*, NM_031885.4; *BBS5*, NM_152384.3; *BBS9*, NM_001033604.1; *C8orf37*, NM_001363260.1; *CEP290*, NM_025114.4; *MC4R*, NM_005912.3; *MKS1*, NM_017777.4. VarSome: P (Pathogenic), LP (Likely pathogenic).

| Patient | Gene | Nucleotide change | Amino acid change | SNP ID | MAF (%) | VarSome |
|---|---|---|---|---|---|---|
| 1 | *BBS1* | c.1169T>G | p.Met390Arg | rs113624356 | 0.1 | LP |
| 2 | *BBS1* | | | | | |
| 3 | *BBS2* | c.1928G>A | p.Arg643His | rs532361142 | 0.004 | LP |
| 4 | *BBS5* | c.551A>G | p.Asn184Ser | rs137853921 | 0.4 | LP |
| 5 | *BBS9* | c.263C>T | p.Ser88Leu | rs749974697 | 0.002 | LP |
| 6 | *C8orf37* | c.374+1G>C | - | rs974031657 | 0.0004 | P |
| 7 | *CEP290* | c.3894dup | p.Lys1299* | rs761907569 | 0.0004 | LP |
| 8 | *MC4R* | c.281G>A | p.Ser94Asn | rs772213710 | 0.0008 | LP |
| 9 | *MCHR1* | c.86C>G | p.Pro29Arg | rs150937708 | 0.006 | LP |
| 10 | *MKS1* | c.1298C>T | p.Thr433Met | rs755841031 | 0.009 | LP |
| 11 | *MKS1* | c.827A>G | p.Asp276Gly | rs151023718 | 0.05 | LP |
| 12 | *MKS1* | c.1349T>C | p.Ile450Thr | rs200865108 | 0.04 | LP |

**Table S4**. Genetic variants identified in candidate genes. MAF = Minor allele frequency in gnomAD. Transcript isoforms: *APOE*, NM_000041.4; *DNAAF1*, NM_178452.6; *ESR1*, NM_000125.3; *GHR*, NM_000163.5; *GUCY2C*, NM_004963.4; *NCOA2*, NM_001321703.2; *NPC1*, NM_000271.5; *PDX1*, NM_000209.4; *RYR1*, NM_*000540.3; STRA6, NM_001199042.2; ZNF423, NM_001271620*.2. VarSome: P (Pathogenic), LP (Likely pathogenic).

| Patient | Gene | Nucleotide change | Amino acid change | SNP ID | MAF (%) | VarSome |
|---|---|---|---|---|---|---|
| 13 | *APOE* | c.305C>G | p.Pro102Arg | rs11083750 | 0.003 | LP |
| 14 | *DNAAF1* | c.715del | p.Ser239AlafsTer12 | rs758650222 | - | P |
| 15 | *ESR1* | c.805C>T | p.Arg269Cys | rs142712646 | 0.09 | LP |
| 16 | | | | | | |
| 17 | | | | | | |
| 18 | *GHR* | c.686G>A | p.Arg229His | rs6177 | 0.1 | LP |
| 19 | | | | | | |
| 20 | *GHR* | c.718T>C | p.Tyr240His | rs143814221 | 0.03 | LP |
| 21 | *GUCY2C* | c.2662del | p.Arg888GlyfsTer4 | rs764325331 | 0.001 | LP |
| 22 | | | | | | |
| 23 | *NCOA2* | c.4366G>T | p.Glu1456Ter | rs1161802201 | 0.0004 | P |
| 24 | *NPC1* | c.665A>G | p.Asn222Ser | rs55680026 | 0.3 | P |
| 25 | | | | | | |
| 26 | *NPC1* | c.3742_3745del | p.Leu1248ValfsTer3 | rs774943545 | 0.003 | P |

| | | | | | | |
|---|---|---|---|---|---|---|
| 26 | *NPC1* | c.2974G>T | p.Gly992Trp | rs80358254 | - | P |
| 27 | *PDX1* | c.97C>A | p.Pro33Thr | rs192902098 | 0.3 | LP |
| 28 | *RYR1* | c.14524G>A | p.Val4842Met | rs193922879 | 0.008 | LP |
| 23 | *RYR1* | c.5602C>G | p.Pro1868Ala | rs780420316 | 0.001 | LP |
| 29 | *RYR1* | c.1807T>C | p.Cys603Arg | rs769492466 | 0.0008 | LP |
| 30 | *RYR1* | c.7025A>G | p.Asn2342Ser | rs147213895 | 0.1 | P |
| 31 | *RYR1* | c.2697C>A | p.Asn899Lys | rs201401814 | 0.004 | P |
| 32 | *STRA6* | c.1684G>A | p.Gly562Ser | rs910950973 | - | LP |
| 33 | *ZNF423* | c.172A>G | p.Met58Val | rs150427822 | 0.006 | LP |

**Table S5**. List of diagnostic and candidate genes for which genetic variants were identified in obese patients. OMIM number, gene-phenotype relationship as from OMIM, OMIM reference, and protein localization as from UniProt are reported for each gene.

| Gene | OMIM | Gene-phenotype relationship | Reference | Protein localization |
|---|---|---|---|---|
| *APOE* | 107741 | Obesity phenotype | 107741 | Extracellular region |
| *BBS1* | 209901 | Bardet-Biedl syndrome 1 | 257220 | BBSome complex |
| *BBS2* | 615981 | Bardet-Biedl syndrome 2 | 606151 | BBSome complex |
| *BBS5* | 615983 | Bardet-Biedl syndrome 5 | 603650 | BBSome complex |
| *BBS9* | 615986 | Bardet-Biedl syndrome 9 | 607968 | BBSome complex |
| *C8orf37* | 614477 | Bardet-Biedl syndrome 21 | 617406 | Base of the primary cilium, cytoplasm |
| *CEP290* | 610142 | Bardet-Biedl syndrome 14 | 615991 | Centriole |
| *DNAAF1* | 613190 | Ciliary dyskinesia, primary, 13 | 613193 | Cilium, cytoplasm, cytoskeleton |
| *ESR1* | 133430 | Estrogen resistance | 615363 | Plasma membrane, nucleus, and cytoplasm |
| *GHR* | 600946 | Laron dwarfism | 262500 | Plasma membrane |
| *GUCY2C* | 601330 | Obesity phenotype | 601330 | Plasma membrane and endosplasmic reticulum |
| *MC4R* | 618406 | Obesity (BMIQ20) | 155541 | Plasma membrane |
| *MCHR1* | 601751 | Obesity phenotype | 601751 | Cell membrane |
| *MKS1* | 609883 | Bardet-Biedl syndrome 13 | 615990 | Basal body |
| *NCOA2* | 601993 | Obesity phenotype | 601993 | Nucleus |
| *NPC1* | 607623 | Niemann-Pick disease, type C1 | 257220 | Endosome and lysosome |
| *PDX1* | 600733 | MODY, type IV | 606392 | Cytoplasm and nucleus |
| *RYR1* | 180901 | Central core disease | 117000 | Sarcoplasmic reticulum |
| *STRA6* | 610745 | Microphthalmia, syndromic 9 | 601186 | Plasma membrane |
| *ZNF423* | 604557 | Impaired adipogenesys | 604557 | Nucleus |

**Table S6**. Deleterious genetic variants identified in clinically followed patients. MAF = Minor allele frequency in gnomAD. Transcript isoforms: *BBS9*, NM_001033604.1; *APOE*, NM_000041.4; *GHR*, NM_000163.5; *PDX1*, NM_000209.4; *ZNF423*, *NM_001271620*.2. VarSome: LP (Likely pathogenic).

| Patient | *Gene* | Nucleotide change | Amino acid change | SNP ID | MAF (%) | VarSome |
|---|---|---|---|---|---|---|
| 5 | *BBS9* | c.263C>T | p.Ser88Leu | rs749974697 | 0.002 | LP |
| 13 | *APOE* | c.305C>G | p.Pro102Arg | rs11083750 | 0.003 | LP |
| 20 | *GHR* | c.718T>C | p.Tyr240His | rs143814221 | 0.03 | LP |
| 27 | *PDX1* | c.97C>A | p.Pro33Thr | rs192902098 | 0.3 | LP |
| 33 | *ZNF423* | c.172A>G | p.Met58Val | rs150427822 | 0.006 | LP |

*Materials and Methods*

While the molecular structures of the regions of interest in BBS9, GHR, and APOE proteins were solved experimentally, we used their respective high-confidence alphafold models [57,58] to make up for the missing residues in the solved structures. The alphafold models were super imposed to the solved structures in PyMOL v2.4.1 [62], and the regions out of our interest were trimmed out from the model. In silico mutagenesis of each variant was performed using the highest-ranked rotamer provided by the "mutagenesis" function of PyMOL [62].
Molecular dynamics simulations were carried out on both wildtype and mutant BBS9, GHR, APOE using Gromacs v2019.3 package [56]. Each protein molecule was placed in a triclinic box with a minimum 1.2 nm spacing on each face. The system was then solvated using TIP3P water molecules, neutralized with $Na^+/Cl^-$ and energy minimized via gradient descent ($F_{max}$ = 100 kJ $mol^{-1}$ $nm^{-1}$, nsteps = 50'000, step_size = 0.001). The minimized systems were subjected to two subsequential equilibration steps position-restrained molecular dynamics in the NVT and NPT ensembles, 100 ps each. Reference temperature of 300 K and pressure of 1 bar were imposed, respectively. Finally, a molecular dynamics production run was performed for 100ns with a 2 fs integration step size in both wildtype and mutants.
The molecular dynamics analysis was performed by first computing the Root Mean Squared Deviation (RMSD) of the backbone atoms, relative to the structure in the minimized and equilibrated system. Highly flexible interdomain loops and termini were excluded from the RMSD due to their large movements during the simulation. The RMSD computation was then followed by Root Mean Squared Fluctuations (RMSF) to compute the fluctuations of the C-$\alpha$ atoms on a per residue basis. To estimate the compactness of the structure during the simulation, the radius of gyration, the number of hydrogen bonds, and the extent of secondary structures were computed.

*Results*

***APOE***

The variant is located in the single β-strand connecting H2 to H3 (the loop's backbone conformations fall into the β region of the Ramachandran plot of the N-terminal domain [59]), and replaces proline with a large charged amino acid – arginine (Figure S1). The mutation causes the region nearby the mutation (the loop comprising residues 98-104) to exhibit larger fluctuations than the wildtype (Figure S2). On the other hand, the other peak that is apparent in the RSMF plot comprises residues 140-150 (which connect H3 to H4 and are responsible for heparin binding), and shows a smaller fluctuation in the mutant [60]. The difference in RMSF concerning the first peak (residues 98-104) is ~0.17 nm, and may in part be linked to a

disruption of the *apoE* N-terminal domain activity. The RMSD scores (Figure S3) and the molecular dynamics movies provide evidence on the stability of both structures throughout the 100ns simulation.
For APOE, the alphafold model was superposed to the structure provided by PDB entry 1BZ4, and the regions out of interest were trimmed out.

***GHR***

RMSD for both structures, oscillated between ~0.1-0.2nm throughout the simulation, providing evidence on the stability of the folded structure (Figure S4).
The p.Tyr240His variant replaces tyrosine (polar non-charged) with histidine (polar charged), introducing a positive charge into a positively charged region (R231, K233) (Figure S5). By using comparative molecular dynamic simulations between the wildtype and mutant, we were able to observe a movement of the loop comprising residues 234-238, in the mutant. In the wildtype, Y240 – which acts as a H-bond acceptor - is part of a hydrogen bonding network (Figure S5), involving E193, R231, and K233, making it stable throughout the 100ns simulation. On the other hand, in the mutant, histidine was not able to form stable hydrogen bonds with the neighboring residues due to the repulsive forces between the positively charged groups of R231, K233 and H240 (Figure S6). The fluctuations observed in residues 234-238 (in proximity to the hGH:receptor interface (PDB entry 1AXI), and including another positively charged residue - R235) (Figure S7) may be a consequence of this variant and may affect the hGH:receptor interface (PDB entry 1AXI). Overall, the introduction of histidine in place of tyrosine 240, despite having similar side chains in terms of size, is able to disrupt the neighboring region due to its positively charged side chain and can alter the ability of the receptor to bind the hGH.
For GHR, the alphafold model was superposed to the structure provided by PDB entry 1AXI, and the regions out of interest were trimmed out.

***BBS9***

The p.Ser88Leu variant replaces serine (polar) with leucine (hydrophobic), and is located in the loop connecting the A and B strands in blade β2 of BBS9 propeller domain, which is in the opposite end of the interface binding to the rest of BBSome complex [61,63]. In the wildtype GLN166 preferres hydrogen bonding with ASP165 and SER88, where the latter acts as a H-bond donor, instead of ARG149 (Figure S8), while in the mutant, the introduction of the hydrophobic LEU88, causes GLN166 to change comformation, and to form two hydrogen bonds with ARG149 (Figure S9). Despite the mutation doesn’t seem to affect the overall folding of the structure, the H-bond network appears to be at least in part disrupted by the introduction of the hydrophobic leucine. The RMSD score of both wildtype and mutant (Figure S10) adheres to an invariant behavior, with fluctuations in the range of ~0.1nm. From the RSMF (Figure S11) plot, two large peaks are observed, corresponding to the loop region connecting the C and D strands of blade β1 and the helix structure to the D strand of blade β4. In the second peak, a difference of ~0.1nm is also observed between the wildtype and mutant (the mutant possessing the larger fluctuations). However, more work is needed to correctly address the pathogenicity of the BBS9 S88L variant at the structural level.
For BBS9, the alphafold model was superposed to the structure provided by PDB entry 4YD8, and the regions out of interest were trimmed out.

**Figure S1.** The superposed structures of wildtype and mutant APOE. The mutant arginine at position 102 is highghlited in orange, and the wildtype proline is highlighted in purple.

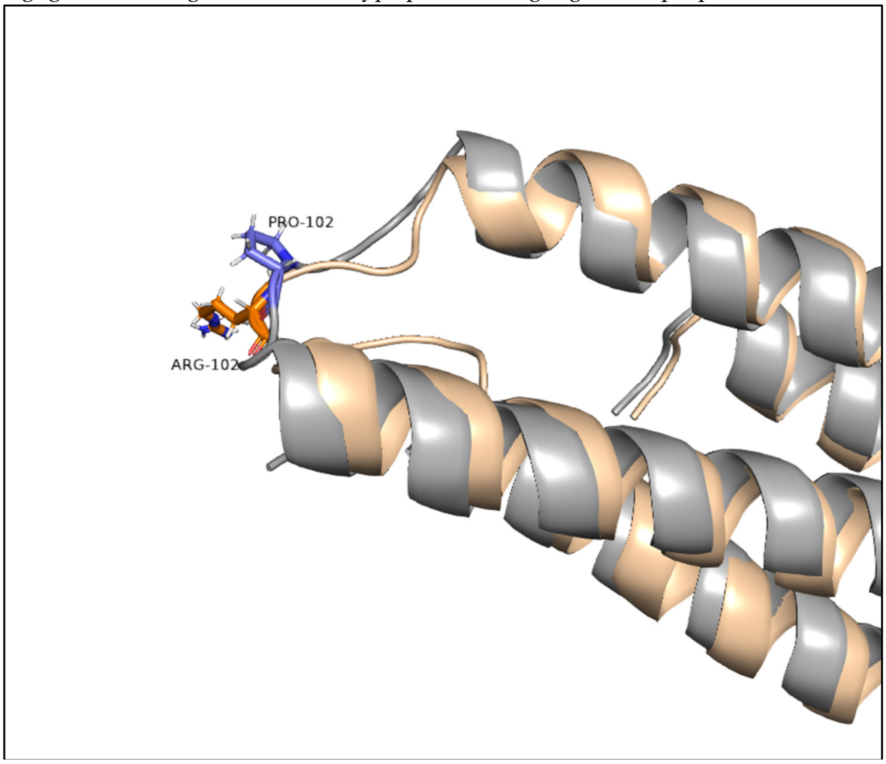


**Figure S2.** RMSF plots of wildtype and mutant APOE.

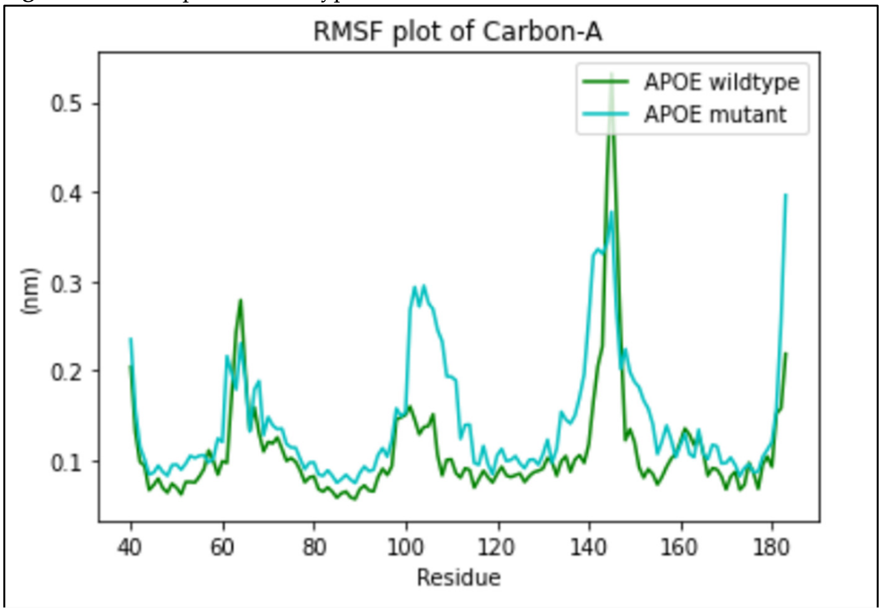

**Figure S3.** RMSD plots of wildtype and mutant APOE.

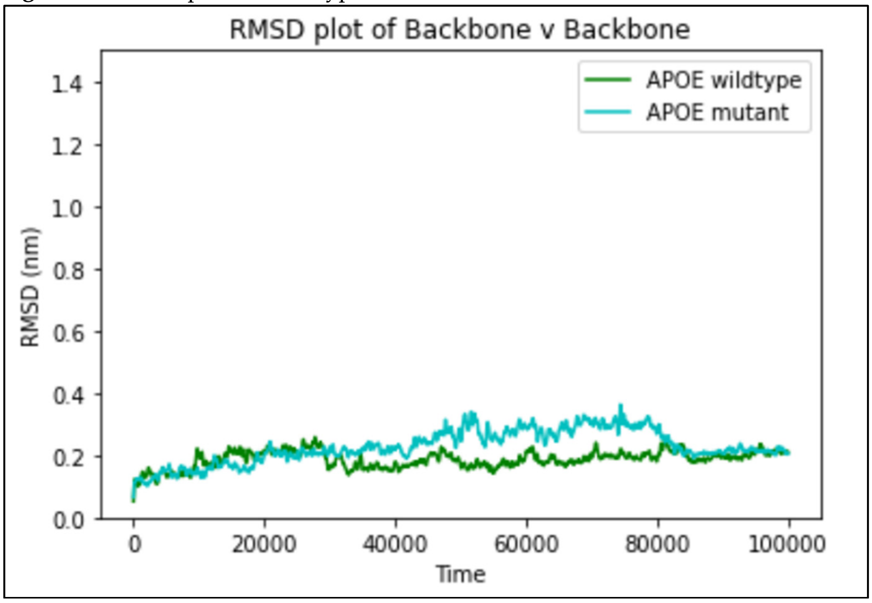


**Figure S4.** RMSD plots of wildtype and mutant GHR.

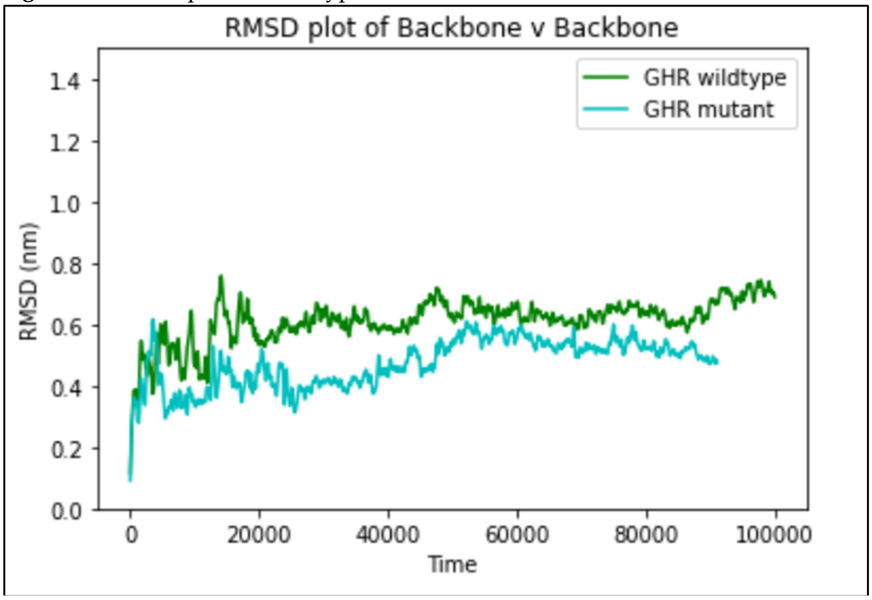

**Figure S5.** Wildtype GHR hydrogen bond network neighboring the mutation site.

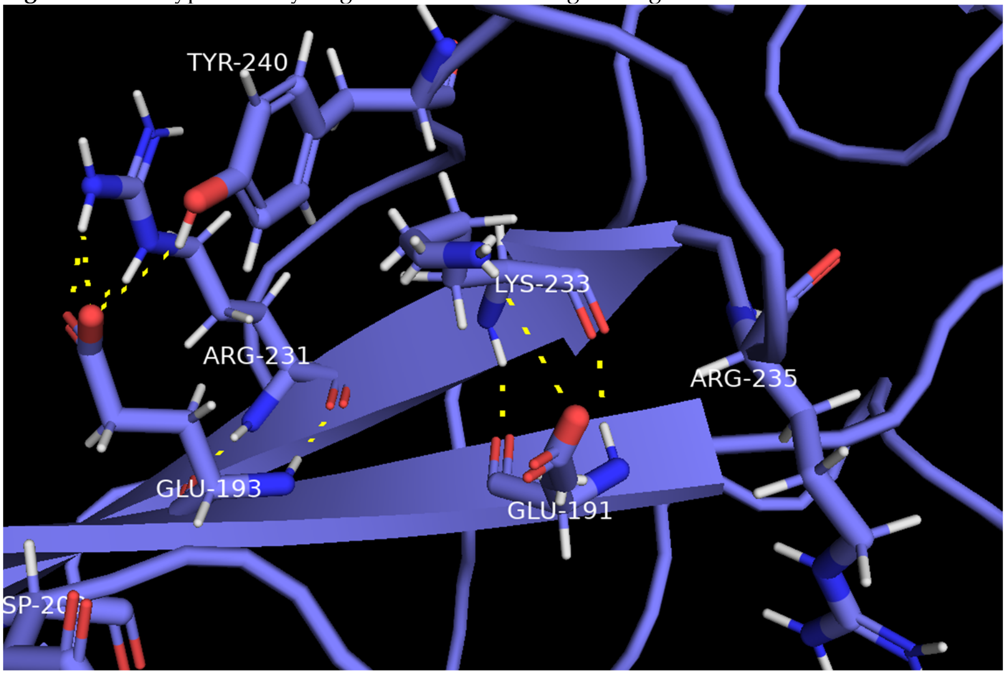


**Figure S6.** Mutant GHR hydrogen bond newtork disruption by the mutant.

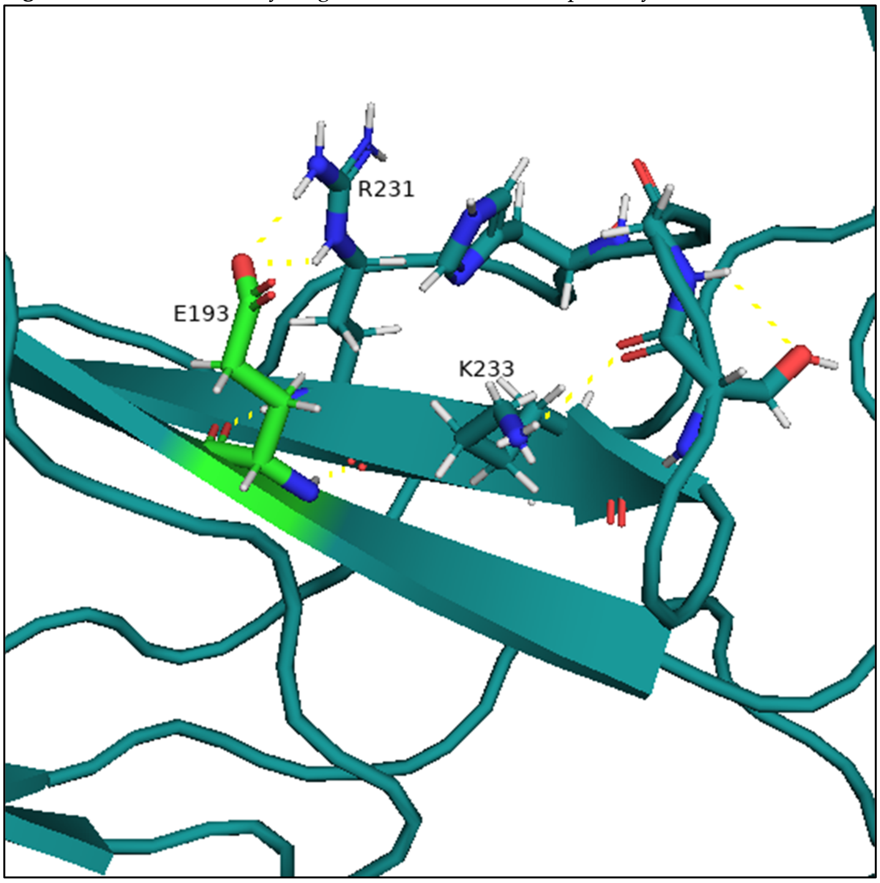

**Figure S7.** RMSF plots of wildtype and mutant GHR.

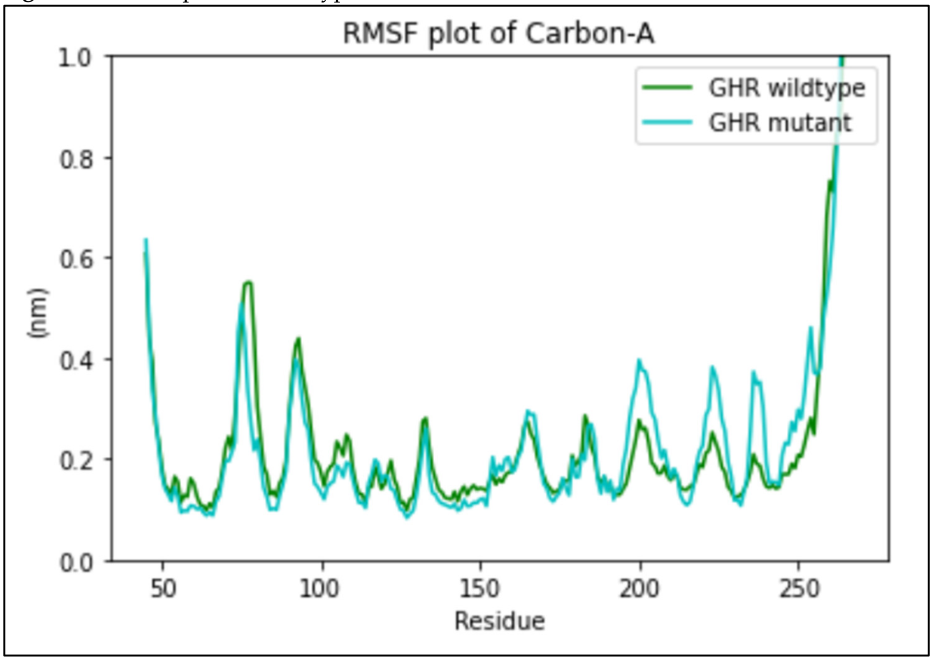


**Figure S8.** Wildtype structure of BBS9, serine 88 and glutamine 166 are highlighted in yellow.

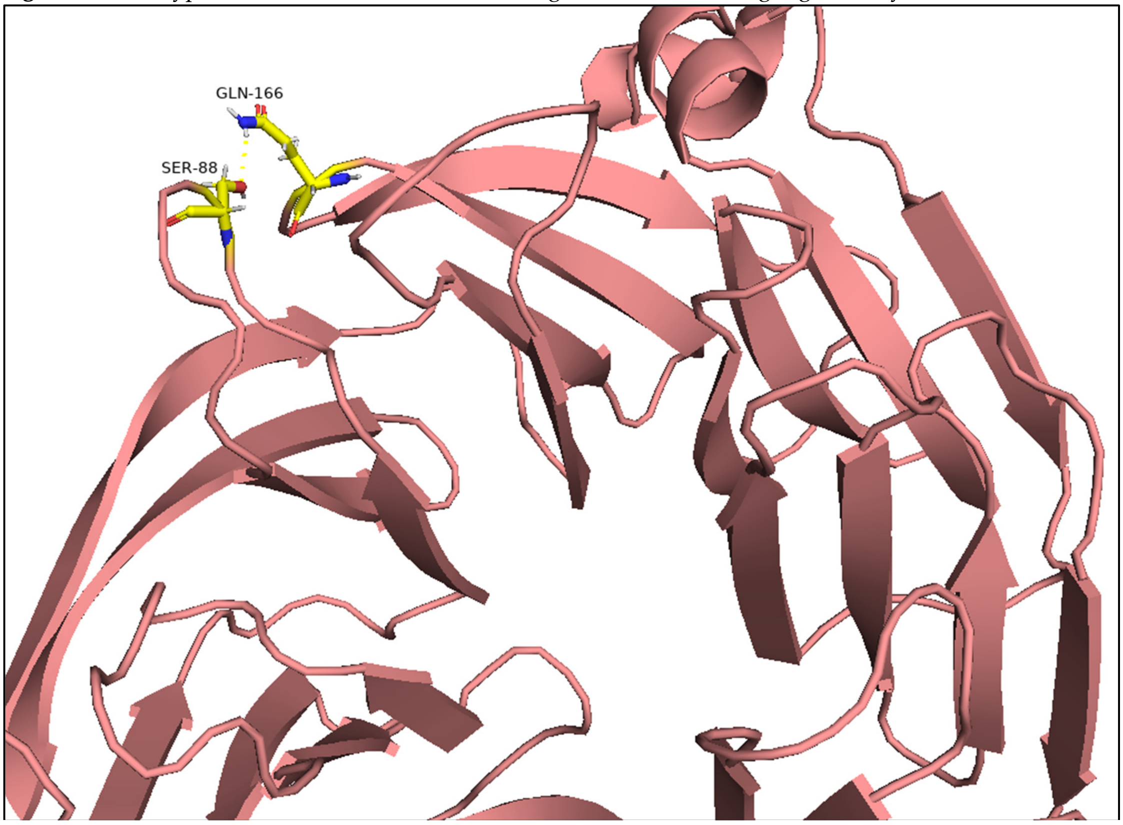

**Figure S9.** Mutant structure of BBS9, serine at position 88 is replaced by leucine, which is hydrophobic and is excluded from the hydrogen bond network formed by serine 88, glutamine 166, and arginine 149.

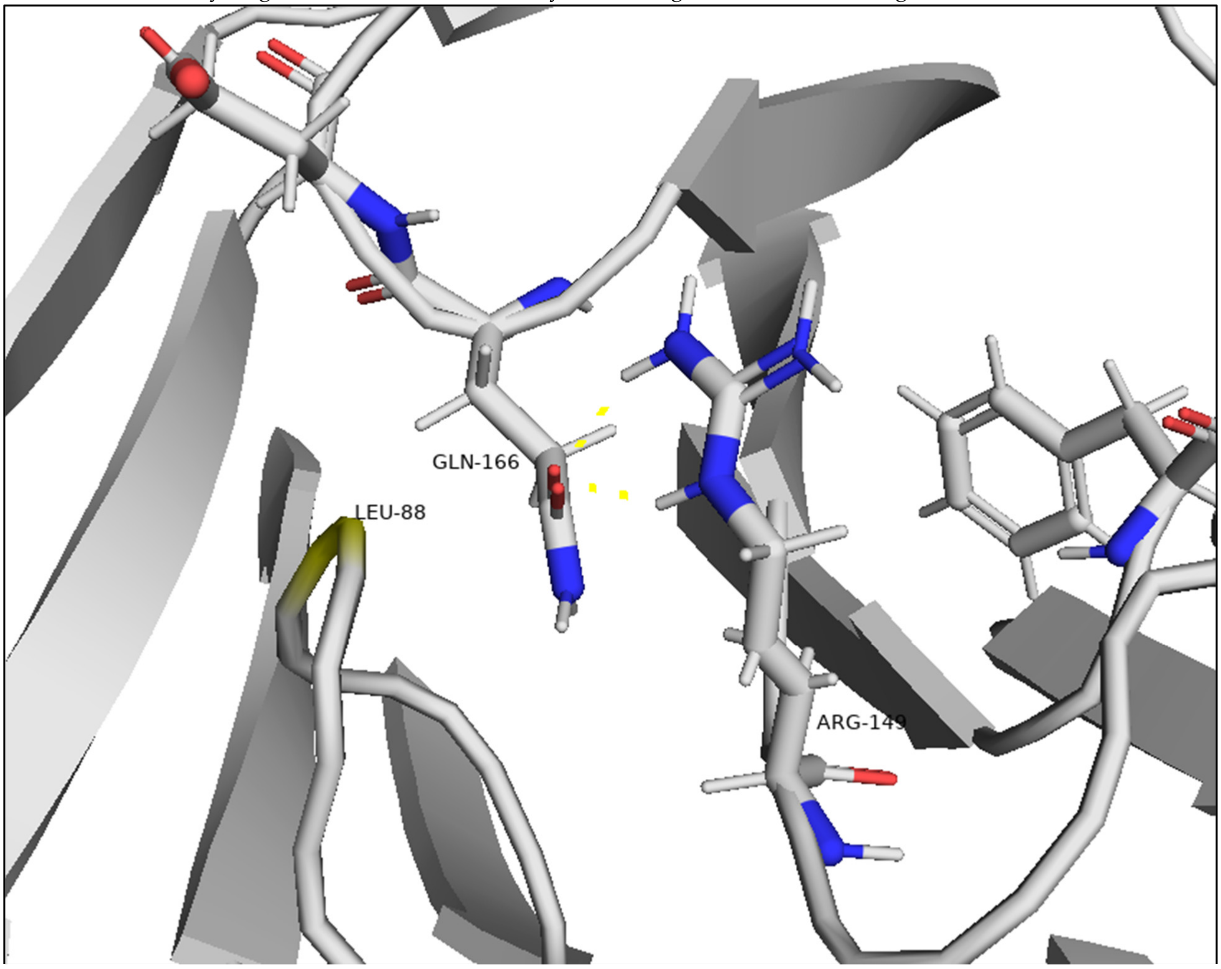

**Figure S10.** RMSD plots of wildtype and mutant BBS9.

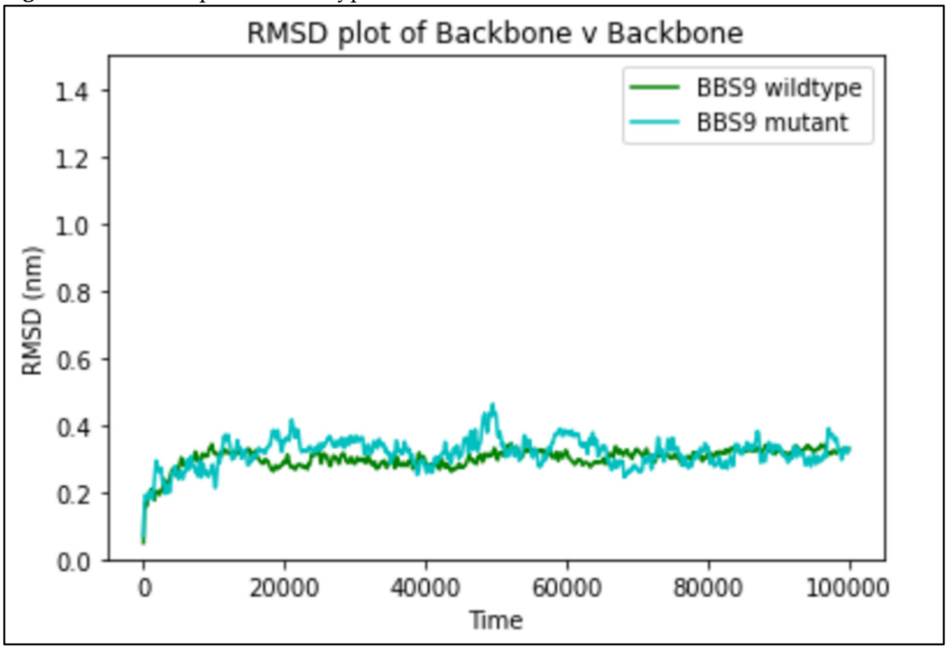


**Figure S11.** RMSF plots of wildtype and mutant BBS9.

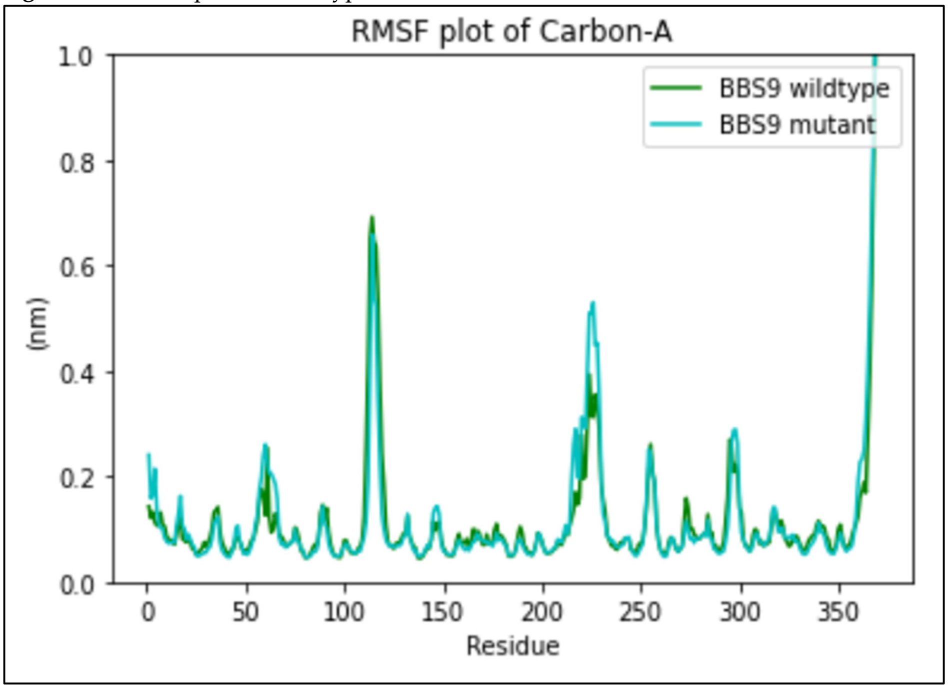

Supplement: Supplementary file 1 [file jcm-11-07531-s001.zip › jcm-2023798-supplementary.pdf]
